# Risk factors for administration of additional neuromuscular block reversal in adults undergoing general anaesthesia: a single centre retrospective case-control study

**DOI:** 10.1186/s12871-025-03009-x

**Published:** 2025-04-17

**Authors:** Susan R. Vishneski, Amit K. Saha, Lan C. Tran, Rohesh J. Fernando, Suneeta K. Acharya, Lisa K. Lee, Leah B. Templeton, Amber K. Brooks, L. Daniela Smith, T. Wesley Templeton

**Affiliations:** 1 Department of Anesthesiology, Wake Forest University School of Medicine, Medical Center Boulevard, Winston-Salem, NC 27157 − 1009 USA; 2 Division of Pediatric Anesthesia, Department of Anesthesiology and Perioperative Medicine, University of California Los Angeles, Los Angeles, CA USA; 3 Atrium Health Carolinas Medical Center, Charlotte, NC USA

**Keywords:** African American, Neostigmine, Rocuronium, Neuromuscular block

## Abstract

**Background:**

Residual neuromuscular block continues to be a modifiable risk factor for major postoperative pulmonary complications in adults.

**Methods:**

We performed a large retrospective case-control study at a single center to evaluate both the prevalence and risk factors for clinically significant residual neuromuscular block following reversal with neostigmine.

**Results:**

We found that clinically significant residual neuromuscular block after reversal with neostigmine is rare, occurring in 3.2% of adults. Risk factors for incomplete reversal with neostigmine following rocuronium administration included: increasing age, ASA physical class status III and IV, a cumulative dose of rocuronium > 0.43 mg•kg^-1^hr^-1^, an interval of < 48 min between the last dose of rocuronium and neostigmine administration, a qualitative train-of-four count < 2 at the time of reversal with neostigmine, emergency case status, thoracic surgery, and African American race.

**Conclusion:**

Reversing neuromuscular block with sugammadex in patients at higher risk of incomplete reversal with neostigmine can improve outcomes and reduce costs, especially in cases where qualitative assessment is utilized or when quantitative monitoring is unavailable.

**Supplementary Information:**

The online version contains supplementary material available at 10.1186/s12871-025-03009-x.

## Background

Residual neuromuscular block (rNMB) following reversal with neostigmine remains a common modifiable contributor to major postoperative pulmonary complications in adults [1, 2]. These complications include serious adverse events such as prolonged post-anesthesia care unit (PACU) stays, aspiration, unplanned reintubation, and pneumonia [3, 4, 5]. Several recent studies have shown that postoperative rNMB can be attributed to variations in provider practice and, in some cases, variability in the pharmacodynamic response to neostigmine and/or sugammadex; although an appropriate dose of sugammadex typically leads to robust reversal of rocuronium or vecuronium [6, 7, 8, 9].

Recently, the American Society of Anesthesiologists (ASA) published practice guidelines that recommend the use of quantitative train-of-four (TOF) monitoring over qualitative assessment to avoid rNMB, and the use of sugammadex over neostigmine at deep, moderate, and shallow depths of neuromuscular block [10]. This may be over-corrective and unrealistic in many practice settings, given the increased cost of quantitative monitoring compared to qualitative assessment and the increased cost of sugammadex compared to neostigmine. Neostigmine has been safely used worldwide since the 1950s for the reversal of neuromuscular blocking agents, including pancuronium, rocuronium, atracurium, and cisatracurium [11]. Further, quantitative monitoring may not be as accurate or robust depending on whether accelerometry- or electromyography-based monitors are utilized [12]. In one study, a quantitative TOF ratio of 0.9 before extubation was not associated with better pulmonary outcomes [13]. Because quantitative TOF monitoring is not yet universally available and sugammadex may not always be accessible due to cost or a possible supply chain issue, the clinician may be able to substantially improve outcomes while reducing cost by judiciously reversing neuromuscular block with neostigmine in settings where the risk of rNMB may be low. This further highlights the need to identify risk factors for rNMB in order to delineate patients who may safely receive neostigmine for reversal from high-risk patients who would benefit from reversal with sugammadex.

Therefore, the primary aim of this study was to evaluate the prevalence and identify potential risk factors associated with rNMB in adults following reversal with neostigmine, using the administration of a rescue dose of sugammadex as a surrogate for rNMB. Further, based on previous research by our group in children, we hypothesized that specific risk factors for rNMB in adults may include ASA physical class statuses III and IV, reduced time between the last dose of neuromuscular block and the administration of reversal, and African American race [14].

## Methods

This was a single center retrospective study conducted at Atrium Health Wake Forest. Atrium Health Wake Forest is a tertiary acute care academic medical center. After local institutional review board approval (IRB00068834), we queried our local electronic health record for patients > 18 years of age who underwent general endotracheal anesthesia from January 1, 2016 to December 31, 2021. This cohort was further refined to patients who received only rocuronium for neuromuscular block and then neostigmine for reversal. Patients who received succinylcholine for intubation but who were then given rocuronium were also included. Patients were excluded if they (1) received more than one type of nondepolarizing neuromuscular block agent, (2) only received sugammadex for reversal, (3) received no reversal agent, (4) remained intubated postoperatively, or (5) were extubated in the PACU. Additionally, we also excluded patients in whom there was not a qualitative TOF count value recorded within 15 min of reversal with neostigmine. This study was designed and reported using the Strengthening the Report of Observational Studies in Epidemiology (STROBE) guideline [15]. This retrospective review was approved by the Wake Forest University Health Sciences Institutional Review Board (IRB00068834), and a waiver of consent was granted given the retrospective nature of the study.

### Primary outcomes and controls

The primary outcome was the administration of sugammadex following initial reversal with neostigmine, which was used as a surrogate for clinically significant rNMB [14]. The control group consisted of patients who were extubated successfully in the operating room following reversal of rocuronium by a single dose of neostigmine. All control cases were electronically reviewed for near term respiratory complications. Near-term complications included reintubation in the PACU following extubation in the operating room, significant desaturation (< 90% SpO_2_ for 3 min or more), administration of additional neostigmine or sugammadex in the PACU, and/or the administration of naloxone or flumazenil. Control cases with near-term respiratory complications were excluded from the analysis.

### Covariates

Demographic data including age, weight, sex, race, procedure type and classification, body mass index (BMI), and ASA physical status were collected from the electronic health record for cases and controls. Race was self-reported by the patients. Surgical procedures were divided into the following categories: abdominal, burn, cardiac, otolaryngology, neurosurgery, non-operating room cases, orthopaedic, plastic, thoracic, urogynecology, vascular, and other. Other covariates included the total cumulative dose of rocuronium in mg/kg/hr, the time interval between the last dose of rocuronium and dose of neostigmine, more than one rocuronium dose administered during the procedure, and the duration of anesthesia. Additional covariates included inpatient versus outpatient status, emergency case status, after-hours cases (defined as any case not performed Monday through Friday between 7 am and 6 pm), neuromuscular disease, end-stage renal disease, and qualitative TOF assessment. The presence of intraprocedural qualitative TOF assessment was defined as the documentation of at least one qualitative assessment of TOF 15 min before the administration of neostigmine. Additionally, the TOF count value, when present, was also examined in the initial univariate analysis. Duration of anesthesia was binned into the 3 categories: < 60 min, 60–120 min, and > 120 min.

### Data validation

5% of cases identified by electronic review as having the outcome of interest were manually reviewed by study staff following data extraction to ensure the accuracy of covariates and the presence of the primary outcome measure.

### Statistical analysis

#### Sample size

An initial query at the start of the project design indicated there might be as many as 1600 cases available. Using 10–15 events per predictor, we estimated that we could develop a model with sufficient precision to assess the association of the predetermined covariates with the outcome of an additional reversal agent. Further, we defined clinical significance as an odds ratio > 1.15 or < 0.85.

A data analysis plan was developed before accessing the data. Patients who received additional reversal were compared with the control group. All categorical covariates were evaluated initially using the Chi-squared test to determine their association with the primary outcome at a univariate level. Where the cell counts were < 5, Fisher’s exact test was used. Continuous covariates were initially evaluated for normalcy using Shapiro-Wilk Normality test and Kolmogorov-Smirnov goodness-of-fit test. Normally distributed data are presented as means with SD, and data that were not normally distributed are presented as medians with interquartile ranges. Normally distributed continuous covariates were compared at the univariate level using an independent, two-sample, two-tailed t-test, while the Mann-Whitney U test was used for the comparison of nonparametric data. The significance of the univariate analysis was established at *P* < 0.05. Prior to execution of the multivariable regression model, the covariates of the cumulative dose of rocuronium and the time interval between the last dose of rocuronium and the dose of neostigmine were dichotomized to maximize sensitivity and specificity using Youden’s index. A multivariable regression model was then created to evaluate the association of all covariates with the administration of additional reversal. The regression model was internally validated using 10-fold, cross-validation to test that the model performed well across randomly selected validation samples from the dataset.

We also performed an additional regression analysis to assess potential confounding in the setting of delayed emergence in which we excluded patients who were extubated with a TOF count of 3 or 4 prior to neostigmine reversal in the intervention group as it would seem rNMB would be significantly less likely in these patients following reversal with neostigmine.

We performed several additional analyses to evaluate potential relationships between race and clinician behavior, as previous research has indicated that discrepancies exist in the use of various anesthetics between patients of a different race [16, 17]. These additional analyses included comparing the time from the last dose of neuromuscular block to reversal, cumulative dose of rocuronium, and the dose of neostigmine between African Americans and all other races combined in patients who were given additional sugammadex. We also compared the prevalence of TOF count values of 0, 1, or 2 between African American patients and non-African American patients closest to the initial reversal. Finally, we analyzed the proportion of patients that received additional sugammadex for a given attending anesthesiologist to determine if one or more attendings were significantly overrepresented in the group with the primary outcome compared to the control group leading to an uninformed selection bias.

Statistical analyses were performed in R v3.6.1 (R Foundation for Statistical Computing) using RStudio environment v1.1.456 (RStudio).

## Results

**Fig. 1:**
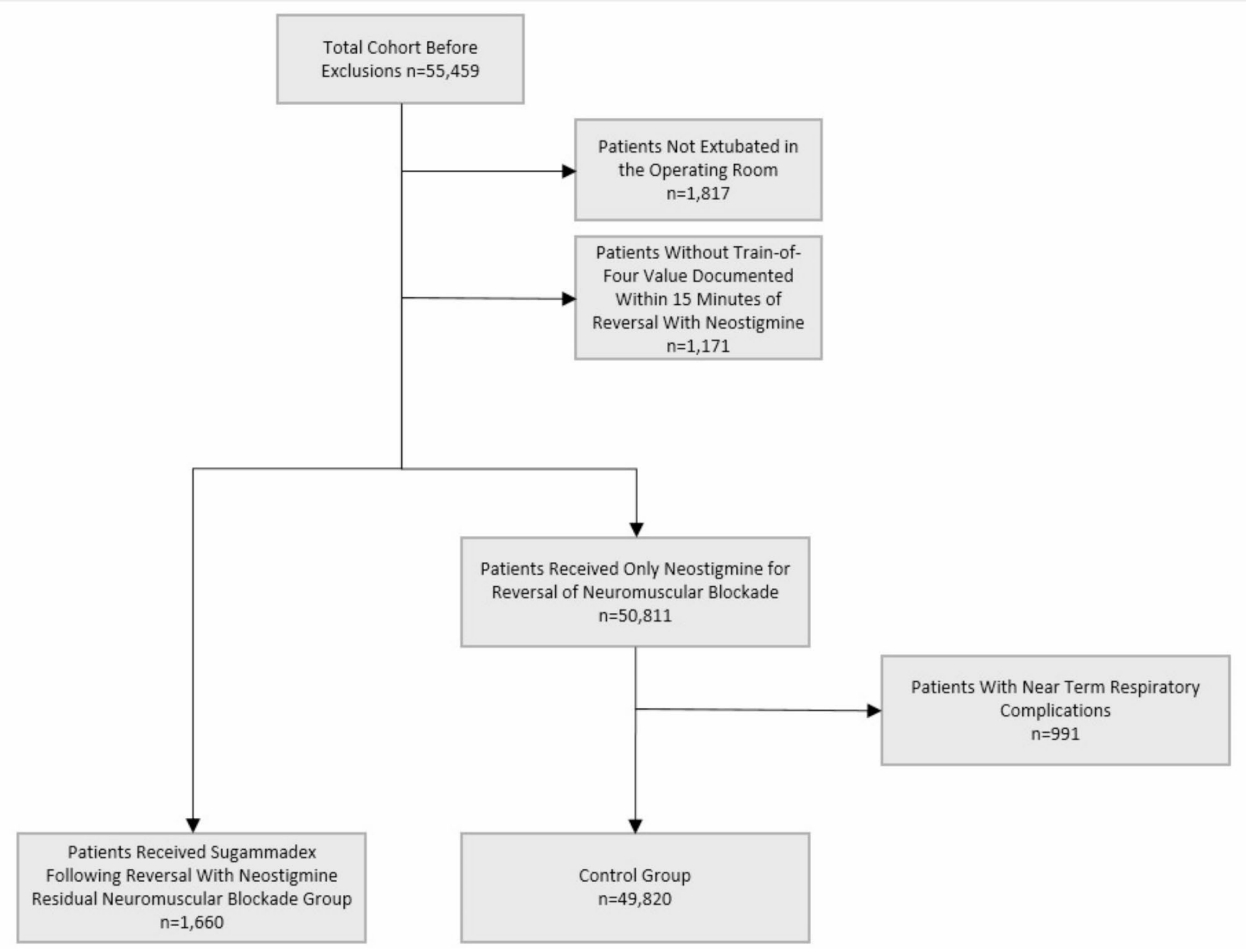
CONSORT diagram of the flow of patients for analysis

A query of our electronic health record revealed 55,459 patients who received a single dose of neostigmine for neuromuscular block reversal following the administration of rocuronium meeting our inclusion criteria between January 1, 2016 and December 31, 2021. Following exclusions for failed extubation in the operating room/PACU, patients who were transported to the intensive care unit (ICU) without being extubated, and patients without TOF assessment within 15 min of reversal, 51,480 patients remained for analysis. Of these, 1660/51,480 (3.2%) patients received additional reversal with sugammadex. The control cohort was further diminished, as 991 patients experienced a short-term study defined pulmonary complication in the operating room or PACU leaving 49,820 control patients. The flow of records available for analysis following exclusions is summarized in Fig. 1.

**Fig. 2:**
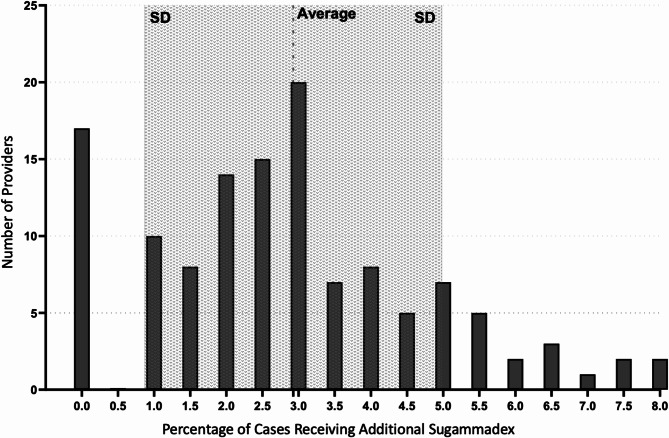
Histogram of case distribution by providers and percentage of cases receiving additional reversal with sugammadex

Maximizing sensitivity and specificity using Youden’s index to dichotomize time from the last dose of rocuronium to the initial dose of neostigmine and cumulative dose of rocuronium, yielded values of 48 min and a cumulative dose of 0.43 mg/kg/hr of rocuronium. A similar Youden’s index analysis using patients that received only a single dose of rocuronium and neostigmine yielded a value of 51 min and a dose of 0.36 mg/kg/hr. The average rocuronium dose in the control group versus the group who received additional reversal was 0.44 mg/kg/hr and 0.64 mg/kg/hr (*P* < 0.001). The median dose of sugammadex rescue was 2.1 mg/kg (IQR 1.8–2.5 mg/kg). Four hundred and twenty-nine of 1660 (25.8%) received sugammadex after extubation. The total number of attending anesthesiologists involved was 111. No one attending anesthesiologist was significantly overrepresented in either the control or the group with the primary outcome. The proportion of patients receiving sugammadex rescue ranged from 0 to 8% for a given attending, with the majority of attending anesthesiologists administering additional sugammadex in 0 to 5% of their cases. This is summarized in Fig. 2. The median time between initial reversal with neostigmine and subsequent administration of sugammadex was 12 min [IQR 8–18 min]. Finally, the median time to successful extubation following administration of a rescue dose of sugammadex prior to extubation was 2 min [IQR 1–4 min].

### Univariate analysis

**Fig. 3:**
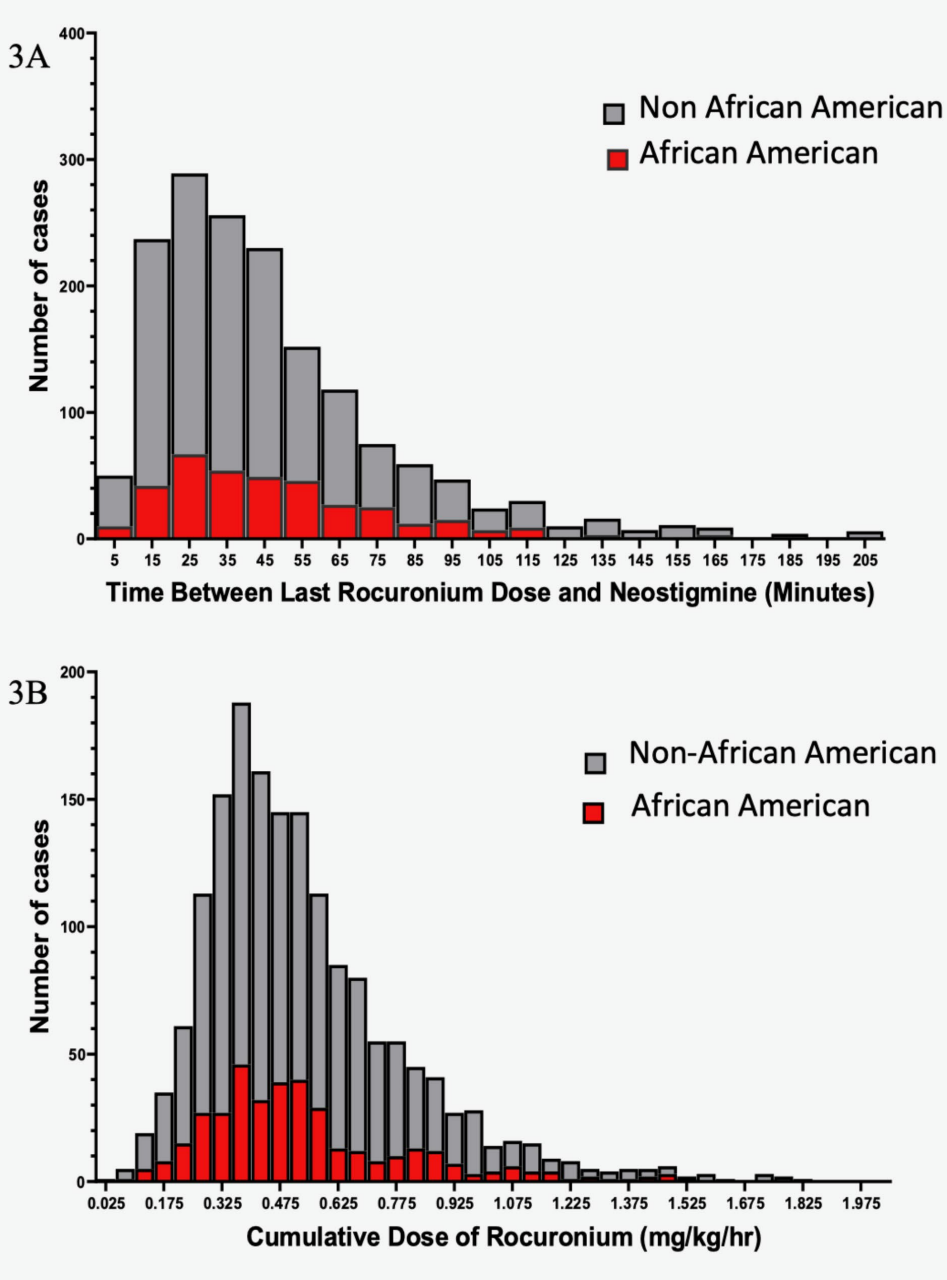
Histogram of cases receiving additional reversal with sugammadex broken down by African American race and all other patients **A**) as a function of time from the last dose of rocuronium and **B**) as a function of cumulative dose of rocuronium (mg/kg/hr)

In the univariate analysis, we found the following factors were significantly associated with the administration of additional reversal: increasing age, BMI, ASA III and IV, inpatient status, case length < 60 min, emergency case, after-hours case, African American race, time between the last dose of rocuronium and reversal administration < 48 min, total cumulative dose of rocuronium > 0.43 mg/kg/hr, and TOF count value < 2. These results are summarized in Table 1. A histogram incorporating African American race and time between the last dose of rocuronium and initial reversal with neostigmine as well as total dose are shown in Fig. 3A and B.

**Table 1:** Demographic data for control group and those receiving a dose of Sugammadex following primary reversal with neostigmine

	Control Group Neostigmine Only *n* = 49,820 *n*, (%)	Neostigmine + Sugammadex *n* = 1660 *n*, (%)	*P* Value
Age (years) (median [IQR])	56.40 [42.20, 67.61]	61.9 [50.9, 71.2]	< 0.001
Body mass index (median [IQR])	28.70 [24.55, 33.23]	30.0 [25.84, 35.4]	< 0.001
**ASA Physical Status**			**< 0.001**
ASA 1 or 2	18,375 (36.9)	272 (16.4)	
ASA 3	27,322 (54.8)	1088 (65.5)	
ASA 4	4123 (8.3)	300 (18.1)	
Inpatient	11,899 (23.9)	564 (34.0)	< 0.001
Emergency case status	6721 (13.5)	395 (23.8)	< 0.001
Female	24,613 (49.4)	777 (46.8)	0.042
Neuromuscular disease	396 (0.8)	22 (1.3)	0.026
After hours cases	2209 (4.4)	135 (8.1)	< 0.001
End-stage renal disease	445 (0.9)	38 (2.3)	< 0.001
**Race**			**< 0.001**
African American	7640 (15.3)	378 (22.8)	
Other	3349 (6.7)	84 (5.1)	
Caucasian	38,831 (77.9)	1198 (72.2)	
Ethnicity Hispanic	2319 (4.7)	55 (3.3)	
Case duration (min) (median [IQR])	175.00 [126.00, 243.00]	165.5[115,249]	0.002
**Surgical Procedure Class**			**< 0.001**
Abdominal/General	12,544 (25.2)	579 (34.9)	
Burn surgery	454 (0.9)	15 (0.9)	
Cardiac surgery	123 (0.2)	7 (0.4)	
ENT surgery	7053 (14.2)	148 (8.9)	
Neurosurgery	2033 (4.1)	68 (4.1)	
Non-operating room anesthesia	1515 (3.0)	58 (3.5)	
Orthopaedic surgery	12,717 (25.5)	282 (17.0)	
Plastic surgery	394 (0.8)	99 (6.0)	
Thoracic surgery	4342 (8.7)	79 (4.8)	
Urogynecologic surgery	1248 (2.5)	251 (15.1)	
Vascular surgery	6194 (12.4)	63 (3.8)	
Other surgical procedures	1203 (2.4)	11 (0.7)	
**Qualitative train of four (TOF)**			**< 0.001**
TOF = 0	7670 (15.4)	161 (9.7)	
TOF = 1	41,887 (84.1)	534 (32.2)	
TOF = 2	7933 (15.9)	390 (23.5)	
TOF = 3	45,676 (91.7)	120 (7.2)	
TOF = 4	4144 (8.3)	455 (27.4)	
Rocuronium redosed	35,036 (70.3)	1289 (77.7)	< 0.001
Rocuronium dose > 0.43 mg/kg/hr	21,901 (44.0)	984 (59.3)	< 0.001
Primary reversal with neostigmine < 48 min after last dose of rocuronium	22,328 (44.8)	1024 (61.7)	< 0.001
Neostigmine dose (mg) (median, [IQR])	4.00 [4.00, 5.00]	5.0 [4.0, 5.0]	< 0.001
Time between neostigmine and extubation (min) (median, [IQR])	10.00 [7.00, 16.00]	13 [9, 19]	< 0.001

ASA, American Society of Anesthesiologists; IQR, interquartile range

### Multivariable analysis

The multivariable regression model demonstrated significant associations with an interval of < 48 min from the last dose of rocuronium to the administration of neostigmine, as well as with a cumulative dose of rocuronium > 0.43 mg/kg/hr. Other factors associated with the administration of additional reversal were increasing age, increasing BMI, ASA III and IV, inpatient status, emergency case, thoracic cases, African American race, and a TOF count < 2. Covariates not associated with the administration of sugammadex included sex, end-stage renal disease, after-hours cases, and case length. These results are summarized in Table 2. A post-*hoc* sensitivity analysis in which we excluded patients in the group that received additional reversal with a TOF count > 2 is presented in Supplemental Table 1.

**Table 2:** Multivariable regression analysis of risk factors for residual neuromuscular block using administration of Sugammadex following primary reversal with neostigmine as a surrogate outcome

	Odds Ratios	95% CI	*P* Value
Age (10-year increments)	1.22	(1.17 to 1.26)	< 0.001
Body mass index (kg/m^2^) (units of 5)	1.21	(1.16 to 1.24)	< 0.001
ASA 1 or 2*	**-**	**-**	**-**
ASA 3	2.02	(1.75 to 2.34)	< 0.001
ASA 4	2.95	(2.43 to 3.58)	< 0.001
Inpatient	1.22	(1.07 to 1.38)	0.002
Emergency case status	1.82	(1.58 to 2.11)	< 0.001
Female	0.92	(0.83 to 1.02)	0.115
Neuromuscular disease	1.39	(0.87 to 2.13)	0.145
After hours cases	1.21	(0.98 to 1.48)	0.071
End stage renal disease	1.36	(0.94 to 1.91)	0.094
Caucasian*	**-**	**-**	**-**
African American	1.64	(1.45 to 1.86)	< 0.001
Hispanic	0.93	(0.64 to 1.35)	0.71
Other	1.04	(0.76 to 1.4)	0.802
Case duration (30-min increments)	1.04	(1.02 to 1.05)	< 0.001
Abdominal/General surgery*	**-**	**-**	**-**
Burn surgery	0.86	(0.48 to 1.42)	0.578
Cardiac surgery	0.67	(0.28 to 1.37)	0.315
ENT surgery	0.67	(0.55 to 0.81)	< 0.001
Neurosurgery	0.63	(0.48 to 0.81)	0.001
Non-operating room anaesthesia	0.77	(0.57 to 1.01)	0.068
Orthopedic surgery	0.6	(0.51 to 0.69)	< 0.001
Plastic surgery	0.66	(0.52 to 0.82)	< 0.001
Thoracic surgery	1.4	(1.08 to 1.79)	0.009
Urogynecologic surgery	1.06	(0.9 to 1.25)	0.458
Vascular surgery	0.81	(0.6 to 1.06)	0.135
Other surgical procedures	0.9	(0.46 to 1.6)	0.746
**Qualitative train of four 2**,** 3**, or 4*	**-**	**-**	**-**
Qualitative train of four = 0 or 1	0.4	(0.36 to 0.45)	< 0.001
Rocuronium redosed	1.11	(0.96 to 1.28)	0.162
Rocuronium dose > 0.43 mg/kg/hr	2.29	(2.02 to 2.59)	< 0.001
Primary reversal with neostigmine < 48 min after last dose of rocuronium	1.43	(1.27 to 1.61)	< 0.001

*=Reference group

ASA, American Society of Anesthesiologists physical status

### Race and additional reversal

The average time from the last dose of rocuronium to the administration of neostigmine reversal in patients of African American race with clinically significant rNMB was 70 ± 56 min versus 67 ± 57 min (*P* < 0.001) in patients of the other race groups combined. The average cumulative dose of rocuronium in African American patients was 1.0 ± 0.9 mg/kg/hr compared to 1.0 ± 1.4 mg/kg/hr (*P* = 0.020) in the other race groups combined, which while statistically significant, is likely not clinically significant. The median TOF count value in the African American group requiring additional reversal was 2/4 versus 2/4 in the non-African American group (*P* = 0.866). An overview of TOF count value data in both the control group and those receiving additional reversal broken down by African American and non-African American race is presented in Table 3. There do not appear to be clinically significant differences in the prevalence of combined 0, 1, or 2 TOF count between African Americans and the other racial groups combined. Finally, the average dose of neostigmine in non-African American patients receiving additional reversal was 0.06 ± 0.001 mg/kg compared to 0.05 ± 0.001 mg/kg (*P* = 0.050) in African American patients. A comparison of African American and non-African American patients is summarized in Table 3.

**Table 3:** Comparison of patient characteristics for African American race and all other races combined for cases in which patients received additional reversal with Sugammadex

	All Others *n* = 1282	African American *N* = 378	*P* Value
Age (years) (median [IQR])	63.1 [51.6, 72.1]	58.2 [48.8, 67.3]	< 0.001
Body mass index (kg/m^2^) (median [IQR])	30.08 [25.92, 35.19]	29.70 [25.66, 36.26]	0.95
ASA 1 or 2, n (%)	209 (16.3)	63 (16.7)	0.886
ASA 3	850 (66.3)	238 (63.0)	0.582
ASA 4	223 (17.4)	77 (20.4)	0.274
Inpatient (%)	429 (33.5)	135 (35.7)	0.453
Emergency case status, n (%)	284 (22.2)	111 (29.4)	0.005
Female, n (%)	602 (47.0)	175 (46.3)	0.867
Neuromuscular disease, n (%)	14 (1.1)	8 (2.1)	0.202
After hours cases, n (%)	94 (7.3)	41 (10.8)	0.037
End stage renal disease, n (%)	24 (1.9)	14 (3.7)	0.058
Case duration (min) (median [IQR])	169 [117, 252]	158 [108, 238]	0.261
Train of four			0.459
0 or 1, n (%)	530 (41.3)	165 (43.6)	
2, 3, or 4	752 (58.7)	213 (56.3)	
**Surgical procedure types**			**0.084**
Abdominal/General surgery	436 (34.0)	143 (37.8)	
Burn surgery	13 (1.0)	2 (0.5)	
Cardiac surgery	6 (0.5)	1 (0.3)	
ENT surgery	117 (9.1)	31 (8.2)	
Neurosurgery	62 (4.8)	6 (1.6)	
Non-operating room anaesthesia	46 (3.6)	12 (3.2)	
Orthopedic surgery	212 (16.5)	70 (18.5)	
Plastic surgery	79 (6.2)	20 (5.3)	
Thoracic surgery	67 (5.2)	12 (3.2)	
Urogynecologic surgery	194 (15.1)	57 (15.1)	
Vascular surgery	43 (3.4)	20 (5.3)	
Other surgical procedures	7 (0.5)	4 (1.1)	
Total rocuronium dose mg/kg/hr	0.48 [0.36, 0.67]	0.48 [0.36, 0.65]	0.876
Rocuronium redosed, n (%)	1012 (78.9)	277 (73.3)	0.024
Rocuronium dose > 0.43 mg/kg/hr (%)	1029 ( 60.0)	228( 60.3)	0.683
Primary reversal with neostigmine < 48 min after last dose of rocuronium	756 (63.3)	212 (56.1)	0.013
Neostigmine dose (mg/kg) (median [IQR])	0.05 [0.05, 0.06]	0.05 [0.04, 0.06]	0.588
Time between neostigmine and sugammadex (min) (median [IQR])	12 [8, 18]	12 [7, 17]	0.103
Time between sugammadex and extubation (min) (median [IQR])	2 [1, 4]	2 [1, 5]	< 0.001

ASA, American Society of Anesthesiologists physical status; IQR, interquartile range

## Discussion

The primary finding of this retrospective case-control study was that clinically significant rNMB after reversal with neostigmine is uncommon, occurring in 3.2% of adult patients within this single center. Notably, the prevalence of clinically significant rNMB within our hospital is much lower than the 56–65% prevalence of rNMB reported in other recent studies where the rNMB was defined as TOF ratio < 0.9 as measured by acceleromyography [6, 9]. A total dose of 0.43 mg/kg/hr and an interval of 48 min between the last dose of rocuronium and neostigmine were also associated with clinically significant rNMB. Other significant findings in our study were the association of increasing age, increasing medical complexity (ASA III and IV), emergency case status, thoracic surgery, and African American race with clinically significant rNMB. Additionally, a qualitative TOF count < 2 within 15 min of reversal was also significantly associated with clinically significant rNMB following reversal with neostigmine. A significant proportion of patients who received additional reversal, 25.8%, received it following extubation.

These findings are clinically important in that they can be used to potentially inform the choice of primary reversal agent in a number of clinical settings. Recent guidelines published by the ASA recommend quantitative monitoring over qualitative assessment, and recommend sugammadex over neostigmine at deep, moderate, and shallow depths of neuromuscular block induced by rocuronium to avoid rNMB [10]. In our control cohort, qualitative TOF assessment was used and 97% of patients were extubated following reversal with neostigmine without additional reversal with sugammadex. It is possible that further investigation of the control group might reveal more subtle pulmonary complications or other sequelae that occurred after leaving the PACU or the hospital in some cases, as 25.9% of the control group patients were discharged to home. Given these findings, it seems difficult to justify requiring all clinicians to use quantitative TOF monitoring and to administer sugammadex over neostigmine considering the significant additional expense and potential, albeit rare, issues such as anaphylaxis associated with the administration of sugammadex [18]. Other complications associated with sugammadex include possible decreased effectiveness of hormonal contraception and increased coagulation parameters. Furthermore, the drug manufacturer’s instructions state that sugammadex is not recommended for use in patients with severe renal impairment, including those requiring dialysis. Therefore, while the findings in our study are not necessarily indicative of causality, they do point to the potential value in using neostigmine for primary reversal in situations associated with a lower risk of inadequate neuromuscular block reversal and sugammadex in settings of higher risk. Additionally, in situations where qualitative TOF assessment is being used, the clinician can use the findings of this study to improve outcomes and potentially reduce cost by using sugammadex as the initial agent in situations associated with a higher risk of incomplete reversal.

Emergency case status and thoracic surgeries were also associated with sugammadex administration after neuromuscular block reversal with neostigmine. These surgeries typically require profound neuromuscular block to optimize [15] operative conditions, as this patient population may be intolerant of the hemodynamic perturbations associated with deep levels of anesthesia required for akinesis. Moreover, these patients are at increased risk for postoperative respiratory complications. Therefore, there are two important considerations in this patient population. First, thoracic patients may be less likely to tolerate any rNMB due to surgery on the chest, which may directly affect the patient’s ability to breathe effectively. Second, clinicians may have a lower clinical threshold for rescue reversal with sugammadex after the failure of initial neuromuscular block reversal with neostigmine [19, 20]. Similarly, increasing age and increasing medical complexity (ASA III and IV) were associated with sugammadex administration after neuromuscular block reversal with neostigmine, which may be due to the inability of these patients to compensate for rNMB as a result of their comorbidities, or perhaps clinician awareness of increased risk for postoperative respiratory complications in these patient populations and thus a lower threshold for rescue reversal [21].

In addition to patient and surgery-specific factors, other risk factors predictive of clinically significant rNMB were a cumulative dose of rocuronium > 0.43 mg/kg/hr and an interval of < 48 min between the last dose of rocuronium and the administration of neostigmine. Previous research from our group has indicated that the interval identified in pediatric patients associated with rNMB was 28 min. Interestingly, the cumulative dose associated with an increased risk of rNMB in children, 0.45 mg/kg/hr was fairly similar to that in adults, 0.43 mg/kg/hr [14]. The increased interval and slightly decreased cumulative dose in adults are consistent with the higher metabolic rate and increased rate of clearance of rocuronium in pediatric patients [22]. Alternatively, children may tolerate incomplete neuromuscular block reversal to a greater extent than their adult and especially elderly counterparts [14].

Finally, the association of African American race with rNMB was unexpected. This finding is, however, congruent with similar findings from the recently published study from our group on the rNMB in pediatric patients [14]. Despite occurring in an entirely different set of patients, undergoing an entirely different group of procedures, with an entirely different group of clinicians, adult African American patients also appear to be at increased risk of clinically significant rNMB like their pediatric counterparts. While the association does not imply causation, the re-demonstration of this finding suggests a potential pharmacogenetic etiology or a more subtle behavioral or dosing-related difference [23–25].

### Limitations

Limitations include the single-center nature of the cohort, although the number of events and robust nature of the control group remains reassuring. In addition, there are inherent limitations related to identification bias and the primary outcome of additional reversal with sugammadex. In some cases, it is possible that additional sugammadex was given in response to a clinical picture that may or may not have been reflective of an actual incomplete reversal. To account for this, we performed a sensitivity analysis, in which patients with a qualitative TOF count of 3 or 4 at the time of reversal with neostigmine were excluded as these patients may be more likely to have been incorrectly identified as requiring additional reversal. The findings in this additional analysis remained fairly unchanged (Supplemental Table 1). The location of qualitative TOF assessment also was not recorded in most cases, and accordingly, there may be differences in the sensitivity of muscle groups to rocuronium which may have impacted the study’s findings [26]. Finally, these results may not be generalizable to patients who have received other nondepolarizing neuromuscular blocking agents or a combination of rocuronium and other nondepolarizing neuromuscular blocking agents.

## Conclusions

In conclusion, clinically significant rNMB was uncommon in our study population of adults after reversal of rocuronium with neostigmine. Risk factors for incomplete reversal of rocuronium following neostigmine administration in adults include: increasing age, ASA III and IV status, emergency case status, thoracic surgeries, African American race, a cumulative dose of rocuronium > 0.43 mg/kg/hr, an interval of < 48 min between the last dose of rocuronium and neostigmine administration, and a qualitative TOF count < 2 at the time of reversal with neostigmine. More prospective research into predictors of rNMB may further aid the clinician in making the optimal choice for reversal. Further, these findings may help improve outcomes in situations where quantitative neuromuscular monitoring is unavailable, or cost constraints or supply chain issues may prohibit the universal use of sugammadex for the reversal of rocuronium.

## Electronic supplementary material

Below is the link to the electronic supplementary material.

Supplementary Material 1

## Data Availability

The datasets used and/or analysed during the current study are available from the corresponding author on reasonable request.

## Abbreviations

ASA: American Society of Anesthesiologists
BMI: Body mass index
ICU: Intensive care unit
PACU: Post-anesthesia care unit
rNMB: Residual neuromuscular block
TOF: Train-of-four
