# Supplementary material for: Risk factors for administration of additional neuromuscular block reversal in adults undergoing general anaesthesia: a single centre retrospective case-control study
Source: BMC Anesthesiol. 2025 Apr 17;25:189. doi: 10.1186/s12871-025-03009-x (PMC12004803; doi:10.1186/s12871-025-03009-x)
Supplement: Supplementary file 1 — Supplementary Material 1 [file 12871_2025_3009_MOESM1_ESM.docx]

**Supplemental Table 1.** Post-*hoc* sensitivity analysis of patients with 3 and 4 qualitative train-of-four count prior to reversal with neostigmine excluded from the intervention group as it seems possible that these patients could have been mis-identified as having residual neuromuscular blockade when in fact there is another explanation for delayed emergence such as excessive sedation.

|  | **Odds Ratios** | **95% CI** | **P Value** |
| --- | --- | --- | --- |
| Age (10-year increments) | 1.22 | (1.17-1.27) | < 0.001 |
| Body mass index (kg/m^2^) | 1.11 | (1.08-1.15) | < 0.001 |
| ASA 1-2* | - | - | - |
| ASA 3 | 2.07 | (1.74-2.47) | < 0.001 |
| ASA 4 | 2.81 | (2.22-3.56) | < 0.001 |
| Inpatient | 1.08 | (0.92-1.26) | 0.351 |
| Emergency | 1.76 | (1.48-2.10) | < 0.001 |
| Female | 1.00 | (0.89-1.14) | 0.946 |
| Neuromuscular disease | 1.44 | (0.81-2.36) | 0.178 |
| End stage renal disease | 1.56 | (1.34-1.83) | < 0.001 |
| After hours cases | 1.14 | (0.87-1.46) | 0.333 |
| **Caucasian*** | **-** | **-** | **-** |
| **African American** | **1.67** | **(1.43-1.93)** | **< 0.001** |
| **Other** | **1.05** | **(0.77-1.42)** | **0.737** |
| **Hispanic** | **0.94** | **(0.59-1.49)** | **0.806** |
| Case duration (30-min increment) | 0.97 | (0.95-0.99) | 0.004 |
| **Abdominal/General surgery*** |  |  |  |
| **Burn surgery** | **0.87** | **(0.41-1.62)** | **0.689** |
| **Cardiac surgery** | **0.89** | **(0.31-2.01)** | **0.798** |
| **ENT surgery** | **0.63** | **(0.50-0.79)** | **<0.001** |
| **Neurosurgery** | **0.77** | **(0.57-1.03)** | **0.0844** |
| **NORA** | **0.67** | **(0.47-0.94)** | **0.0261** |
| **Orthopedic surgery** | **0.53** | **(0.44-0.63)** | **<0.001** |
| **Plastic surgery** | **0.55** | **(0.41-0.73)** | **< 0.001** |
| **Thoracic surgery** | **0.93** | **(0.64-1.31)** | **0.694** |
| Urogynecologic surgery | **0.93** | **(0.76-1.12)** | **0.440** |
| **Vascular surgery** | **0.77** | **(0.53-1.07)** | **0.131** |
| **Other surgical procedures** | **0.77** | **(0.326-1.54)** | **0.507** |
| Rocuronium redosed | 0.67 | (0.55-0.82) | < 0.001 |
| Rocuronium dose > 0.68 mg/kg/hr | 2.32 | (1.88-2.88) | < 0.001 |
| Primary Reversal with Neostigmine < 48 min after last dose of rocuronium | 1.56 | (1.34-1.83) | < 0.001 |

*Reference group

ASA, American Society of Anesthesiologists physical status; NORA, Nonoperating room anesthesia
